# Type of anesthesia for cancer resection surgery: No differential impact on cancer recurrence in mouse models of breast cancer

**DOI:** 10.1371/journal.pone.0293905

**Published:** 2023-11-27

**Authors:** Julia Dubowitz, Alexandra I. Ziegler, Richard Beare, Fabian Jost-Brinkmann, Adam K. Walker, Ryan D. Gillis, Aeson Chang, Ni-Chun Chung, Olga A. Martin, Frédéric Hollande, Bernhard Riedel, Erica K. Sloan

**Affiliations:** 1 Drug Discovery Biology Theme, Monash Institute of Pharmaceutical Sciences, Monash University, Parkville, Victoria, Australia; 2 Division of Cancer Surgery, Department of Anaesthesia, Peter MacCallum Cancer Centre, Melbourne, Victoria, Australia; 3 Centre for Integrated Critical Care, Melbourne Medical School, University of Melbourne, Melbourne, Victoria, Australia; 4 Peninsula Clinical School, Monash University, Melbourne, Victoria, Australia; 5 Developmental Imaging, Murdoch Children’s Research Institute, Melbourne, Victoria, Australia; 6 Department of Hepatology and Gastroenterology, Charité –Universitätsmedizin, Berlin, Germany; 7 Freie Universität Berlin and Humboldt-Universität zu Berlin, Berlin, Germany; 8 Neuroscience Research Australia, Randwick, New South Wales, Australia; 9 Discipline of Psychiatry and Mental Health, University of New South Wales, Randwick, New South Wales, Australia; 10 Sir Peter MacCallum Department of Oncology, The University of Melbourne, Melbourne, Victoria, Australia; 11 Centre for Medical Radiation Physics (CMRP), Faculty of Engineering and Information Sciences, University of Wollongong, Wollongong, New South Wales, Australia; 12 Department of Clinical Pathology, The University of Melbourne, Melbourne, Victoria, Australia; 13 The University of Melbourne Centre for Cancer Research, Melbourne, Victoria, Australia; University of Michigan Medical School, UNITED STATES

## Abstract

**Background:**

Surgery is essential for curative treatment of solid tumors. Evidence from recent retrospective clinical analyses suggests that use of propofol-based total intravenous anesthesia during cancer resection surgery is associated with improved overall survival compared to inhaled volatile anesthesia. Evaluating these findings in prospective clinical studies is required to inform definitive clinical guidelines but will take many years and requires biomarkers to monitor treatment effect. Therefore, we examined the effect of different anesthetic agents on cancer recurrence in mouse models of breast cancer with the overarching goal of evaluating plausible mechanisms that could be used as biomarkers of treatment response.

**Methods:**

To test the hypothesis that volatile anesthesia accelerates breast cancer recurrence after surgical resection of the primary tumor, we used three mouse models of breast cancer. We compared volatile sevoflurane anesthesia with intravenous propofol anesthesia and used serial non-invasive bioluminescent imaging to track primary tumor recurrence and metastatic recurrence. To determine short-term perioperative effects, we evaluated the effect of anesthesia on vascular integrity and immune cell changes after surgery in animal models.

**Results:**

Survival analyses found that the kinetics of cancer recurrence and impact on survival were similar regardless of the anesthetic agent used during cancer surgery. Vascular permeability, immune cell infiltration and cytokine profiles showed no statistical difference after resection with inhaled sevoflurane or intravenous propofol anesthesia.

**Conclusions:**

These preclinical studies found no evidence that choice of anesthetic agent used during cancer resection surgery affected either short-term perioperative events or long-term cancer outcomes in mouse models of breast cancer. These findings raise the possibility that mouse models do not recapitulate perioperative events in cancer patients. Nonetheless, the findings suggest that future evaluation of effects of anesthesia on cancer outcomes should focus on cancer types other than breast cancer.

## Introduction

More than 60% of all cancer patients require surgical resection of their cancer [1]. An additional 20% of patients may require anesthesia for diagnostic or supportive care procedures (for example, for imaging or port insertion) or for other cancer therapies (such as high density radiation), thereby exposing up to 80% of cancer patients to anesthetic agents. Two techniques are commonly used to administer general anaesthesia: propofol-based total intravenous anaesthesia (TIVA) and inhalational volatile-based anaesthesia. Both modalities have a robust safety profile, and choice for their use depends on patient characteristics, surgical factors and anaesthetist preferences. A recent survey of Australian and New Zealand anaesthetists found that volatile anaesthesia was the preferred choice due to increased familiarity with the technique [2]. However, several retrospective analyses raise the possibility that use of volatile anesthesia during cancer resection surgery may adversely impact cancer recurrence and overall survival [3–8]. In a retrospective analysis of 5,214 propensity-matched patients with diverse cancer types, use of volatile anesthesia was associated with a significant reduction in overall survival after cancer surgery compared to propofol-based total intravenous anesthesia (TIVA) [3]. Similar findings have reported in some analyses of breast cancer patients [4, 5, 8] although not all [9], and from a number of studies in other cancer types [6, 7]. However, this finding is yet to be confirmed in large scale, prospective well-controlled clinical trials.

It is conceivable that anesthetic agents may impact multiple aspects of the metastatic cascade to impact cancer recurrence. During surgery, tumor cells are released from the tumor into blood and lymphatic circulation [10]. Although few tumor cells survive the shear stress in blood circulation [11], volatile anesthetic agents may activate survival pathways in tumor cells, while propofol may activate apoptotic pathways that could eliminate tumor cells released into circulation [12–14]. Volatile anesthesia also could increase colonization of metastatic target organs by increasing vascular permeability or increasing tumor cell retention [15]. Volatile anesthetic agents have been shown to act on brain endothelial cells to increase blood-brain-barrier permeability [16], but the effect on vasculature permeability in peripheral organs that may aid tumor cell extravasation is unknown. Volatile anesthesia may also modulate anticancer immunity by impairing natural killer cell function [17] and increasing inflammatory cytokines [18]. These findings suggest that volatile anesthesia may amplify anti-apoptotic, pro-angiogeneic, and immune-modulating wound healing responses that characterize the perioperative period [19], while propofol has been shown to have anti-inflammatory and anti-oxidative effects [14, 20].

To explore the impact of anesthesia on cancer recurrence, we set out to model the findings of retrospective clinical studies in breast cancer patients that suggested there may be differential effects of inhalational volatile anesthesia versus propofol-based total intravenous anesthesia on long-term cancer outcomes [4, 8, 9]. Using three well-characterized mouse models of breast cancer progression [21–23], we investigated the effect of anesthesia with sevoflurane or propofol-based total intravenous anesthesia on primary tumor recurrence and metastasis onset and development. Given that volatile anesthesia may also modulate adaptive anti-cancer immunity [18, 24, 25], we hypothesized that a differential effect of anesthetic drug on recurrence may require an intact immune system. Therefore, this evaluation of recurrence after primary tumor resection included two immune-competent models of breast cancer. To further understand the effect of anesthetic agents on short term consequences of surgery, we investigated the effect of anesthesia agents on vascular integrity and the immune response immediately after cancer resection surgery.

## Materials and methods

### Animals

All procedures involving mice were carried out under protocols approved by the Institutional Animal Ethics Committee (MIPS-2015-13121, Monash University, Australia) and in accordance with guidelines of the National Health and Medical Research Council, Australia. Female BALB/c nu/nu (Animal Resources Centre, Western Australia) and BALB/c mice (Monash University, Australia) were housed in specific pathogen free cages in a temperature and humidity-controlled environment with a 12h light-dark cycle. Food and water were available *ad libitum*. Mice were acquired at 6–8 weeks old and were given one week to acclimate before experimentation commenced. Humane endpoints based on body weight loss, body condition score and metastatic progression tracked by non-invasive bioluminescence imaging were used. Mice were euthanized with CO_2_. No unexplained mortality occurred in these studies.

### Cell culture

The human metastatic breast cancer cell line MDA-MB-231^HM^ was a kind gift from Dr Zhou, Fudan University, Shanghai Cancer Centre, China. It is described as MDA-MB-231 throughout the manuscript. The mouse mammary cell lines 4T1.2 (highly metastatic) and 66cl4 (low metastatic) were a kind gift from Prof Robin Anderson, Olivia Newton John Cancer Research Institute, Australia. MDA-MB-231 and 4T1.2 were cultured in Dulbecco’s Modified Eagle’s medium-GlutaMAX media (Life Technologies), and 66cl4 was cultured in alpha-Modified Eagle’s Medium-GlutaMAX media (Life Technologies). All media was supplemented with 10% fetal bovine serum (Life Technologies). Cancer cell lines were maintained at 37°C and 5% CO_2_ and confirmed to be negative for mycoplasma [26]. Cell line identities were confirmed by short tandem repeat profiling (Cellbank, Australia). Tumor cells were stably transduced to express codon-optimized firefly luciferase under control of the ubiquitin-C promoter [27].

### Mouse breast cancer surgery models

Tumor cells (1 × 10^5^ 66cl4 or 4T1.2, 2 × 10^5^ MDA-MB-231) in 20 μL Dulbecco’s Phosphate Buffered Saline (Invitrogen) were injected into the 4^th^ inguinal mammary fatpad of anesthetized (3–5% isoflurane) female mice. Primary tumor growth was monitored by digital caliper twice per week and tumor volume calculated using the formula (length × width^2^)/_2_. The primary tumor was resected under the randomized anesthetic technique (see below) prior to the onset of overt metastasis: primary tumor volume: ~90 mm^3^ for MDA-MB-231, ~150 mm^3^ for 66cl4 or ~60 mm^3^ for 4T1.2. For resection, a 1cm incision was made inferior to the tumor in the region of the left fourth mammary fatpad and the tumor and adjacent left inguinal lymph node were resected. Clear surgical margins were confirmed by bioluminescence imaging with injection of *d*-luciferin (150 mg.kg^-1^, Promega) in 100 μL Dulbecco’s Phosphate Buffered Saline via tail vein, and bioluminescence from any residual tumor cells was quantified using an IVIS Lumina II (Perkin Elmer) with Living Image Software. The skin was closed with either 5–0 nylon sutures or sterile wound clips. Surgery was conducted in a thermoregulated environment using a heating pad. Buprenorphine (0.05 mg/kg) was injected subcutaneously for analgesia before surgery and every 12h after surgery for 48h.

Stress response signaling through neural and inflammatory pathways are evolutionarily conserved in mice and humans. However, compared to humans, rodents are resistant to inflammatory challenge [28]. Therefore, to induce an inflammatory response of similar magnitude to that induced by surgery in cancer patients, tumor resection was accompanied by midline laparotomy with externalization of bowel [29]. The externalized bowel was covered with a saline-soaked gauze for 30 min before being re-internalized, the peritoneum closed with absorbable sutures followed by the external skin using nylon sutures. We confirmed that compared to unoperated mice or mice in which the primary mammary tumor and surrounding fatpad only were resected, inclusion of the laparotomy at the time of mammary tumor removal increased the inflammatory response, as seen by enlarged spleen mass and elevated cytokine levels (S1 Fig S1 File).

### Anesthesia technique

On the day of surgery, mice were randomly assigned to surgical resection under two different anesthetic techniques. For the volatile condition, anesthesia was maintained with 3–5% sevoflurane (Baxter), adjusted as required to maintain a stable depth of anesthesia (loss of corneal and pedal reflex, respiratory rate less than 100 breaths per minute). For the intravenous propofol condition, after brief induction with 3–5% sevoflurane, the lateral tail vein was catheterized and 2% Propofol-Lipuro (Braun) was administered with an initial bolus of 27 mg/kg over 60 seconds. The sevoflurane administration was then halted and the propofol infusion continued at a rate of 2.2–4.0 mg/kg/min to maintain stable depth of anesthesia, as described above. All mice received anesthetic exposure for one hour, during which time the surgery was performed. Thereafter, mice were allowed to wake up in a clean cage placed on a heating pad until normal alertness and were then returned to their home cage.

### Tumor recurrence

Mice were monitored for primary tumor recurrence and onset of metastasis twice a week using bioluminescence imaging by an investigator blinded to the assigned anesthesia condition used during surgical resection. Mice were injected with *d*-luciferin (150 mg/kg, Promega) in 100 μL Dulbecco’s Phosphate Buffered Saline via tailvein under anesthesia and tumor bioluminescence was quantified using an IVIS Lumina II (Perkin Elmer) with Living Image Software. Recurrence after primary tumor resection was analyzed over seven individual experimental replicates, each with 7–9 mice per condition: four replicates with MDA-MB-231 (total n = 33–34 mice), two replicates with 66cl4 (n = 15–17 mice) and a single replicate with 4T1.2 (n = 7–9 mice).

### Vascular integrity

Sodium fluorescein (100 μl of 100 mg/ml, Sigma-Aldrich) was injected intravenously through the tail vein. Thirty minutes later plasma was obtained from cardiac blood before perfusion with phosphate buffered saline to remove circulating blood and fluorescein from tissue vasculature. The lung was dissected, homogenized in phosphate buffered saline and centrifuged at 10,000 g for 5 min to obtain supernatant. Lung supernatant and plasma were diluted with phosphate buffered saline and sodium fluorescein was quantified within a linear range of standards of known concentration using a fluorescent microplate reader, with excitation at 493 nm, emission wavelength of 538 nm, and auto cutoff for the high-pass filter.

### Immune cell profiling

Single cell suspensions from freshly dissected spleens and lungs were obtained by gently pressing samples through a 40 μm cell strainer. Red blood cells were removed using red blood cell lysing buffer (Sigma-Aldrich). Cells (3 × 10^6^) were incubated with antibodies (below) for 30 min at 4°C, washed and resuspended in 2% fetal bovine serum in Dulbecco’s Phosphate Buffered Saline, and analyzed using a Stratedigm S1000EXi. Data were analyzed using FlowJo10 (Tree Star Inc.) software.

The following antibodies from BD Pharmingen, eBioscience and Biolegend were used for staining: Phycoerythrin (PE) cyanine dye (Cy) PECy7-conjugated CD49b, PECy7-CD11b, Fluorescein-isothiocyanate (FITC)-conjugated Ly6G, FITC-Ly6G, FITC-CD4, Bright Violet (BV) BV710-conjugated Ly6C, BV785-CD11b, BV650-CX3CR1, BV570-CD8, BV421-CD11c, Alexa Flour (AF) conjugated AF700-CD25, allophycocyanin (APC) APC-F4/80, APC-CD44, APCCy7- MHCII, APC-Cy7-CD69, BD Horizon™ V450-conjugated V450-CD11c, V500-CD8, eF660 F4/80, eF450-CD62L. Propidium Iodide was used to detect dead cells.

For the intracellular staining of CD4+CD25+FoxP3+ regulatory T cells the FoxP3/Transcription Factor Staining Buffer Set (eBiosciences) was used according to the manufacturer’s instructions.

### Cytokine analysis

Cytokine levels were measured using BioPlex Pro™ mouse cytokine 23-plex kit (Bio-Rad, Gladesville, Australia) according to the manufacturer’s protocol using a BioPlex200 machine (BioRad). Lung or plasma were analyzed 24h after resection of 66cl4 tumors. Median fluorescence data was collected and analyzed using BioPlex Manager software using a 5-parameter logistic (5-PL) method.

### Statistical analysis

The effect of anesthetic type on cancer progression was assessed by examining the effect on both the onset and the kinetics of cancer recurrence. Survival analyses were used to explore group differences (sevofluorane vs. propofol) in time taken to primary tumor recurrence and time to metastasis (distant recurrence). S1 Table (see S1 File) describes the groups that were compared and the total number of mice (experimental unit) in each group. No mice were excluded from the analysis. No prior information was available on effect size. A Bayesian multi-level approach was used to assess group differences in the kinetics of primary tumor recurrence and metastasis development, in cases where recurrence of the primary tumor and/or metastasis occurred.

Two forms of survival analysis were performed. First, Kaplan-Meier survival models were used to examine each mouse cancer model separately (MDA-MB-231, 66cl4, and 4T1.2). Mice in which recurrence and/or metastasis were not observed were considered censored. The second approach used a multilevel Bayesian regression to maximise power by pooling data across all replicates from all three mouse cancer models. The Bayesian regression framework minimised dependence on parametric distributions, allowed inclusion of a censored response and supported a nested model of the experiment structure to allow for differences in error distributions between batches and mouse types. Time to recurrence was the dependent variable and anesthesia type was the independent variable in the Bayesian regression model. The model assumed a censored log-normal response distribution and used a random effect of experimental replicate nested within mouse cancer model type to model experimental structure. Priors were minimally informative: Student-t priors, degree of freedom = 3, SD = 10.

A pooled analysis, also using a Bayesian multilevel approach, was used to test differences between kinetics of primary tumor recurrence due to the low numbers of recurrences per experimental replicate. The dependent variable was log of bioluminescent flux (detecting tumor recurrence) and the independent variable was time since recurrence. A random effect of mouse identifier was included in addition to the nested groups described above to account for multiple measures per mouse over time. The effect of anaesthesia type was assessed via a group interaction term.

Bayesian credibility intervals (CI) at the 95% level were used to evaluate the estimated group differences in survival time and recurrence rates, with a CI crossing zero indicating that a difference cannot be detected. Kaplan-Meier survival analysis and Bayesian regression were performed with the *survival* and *brms* packages for R 3.5 [30–32]. Differences in immune cell populations were tested using nonparametric Wilcox tests and false discovery rate was used to correct for multiple comparisons. Differences in vascular permeability were tested using an analysis of variance adjusted for experimental replicate batch. Statistical analyses were undertaken by an investigator (RB) blinded to treatment conditions. All data included in the manuscript are available at DOI: 10.5281/zenodo.8429174.

## Results and discussion

### Cancer recurrence in a xenograft model of human breast cancer

**Fig 1:**
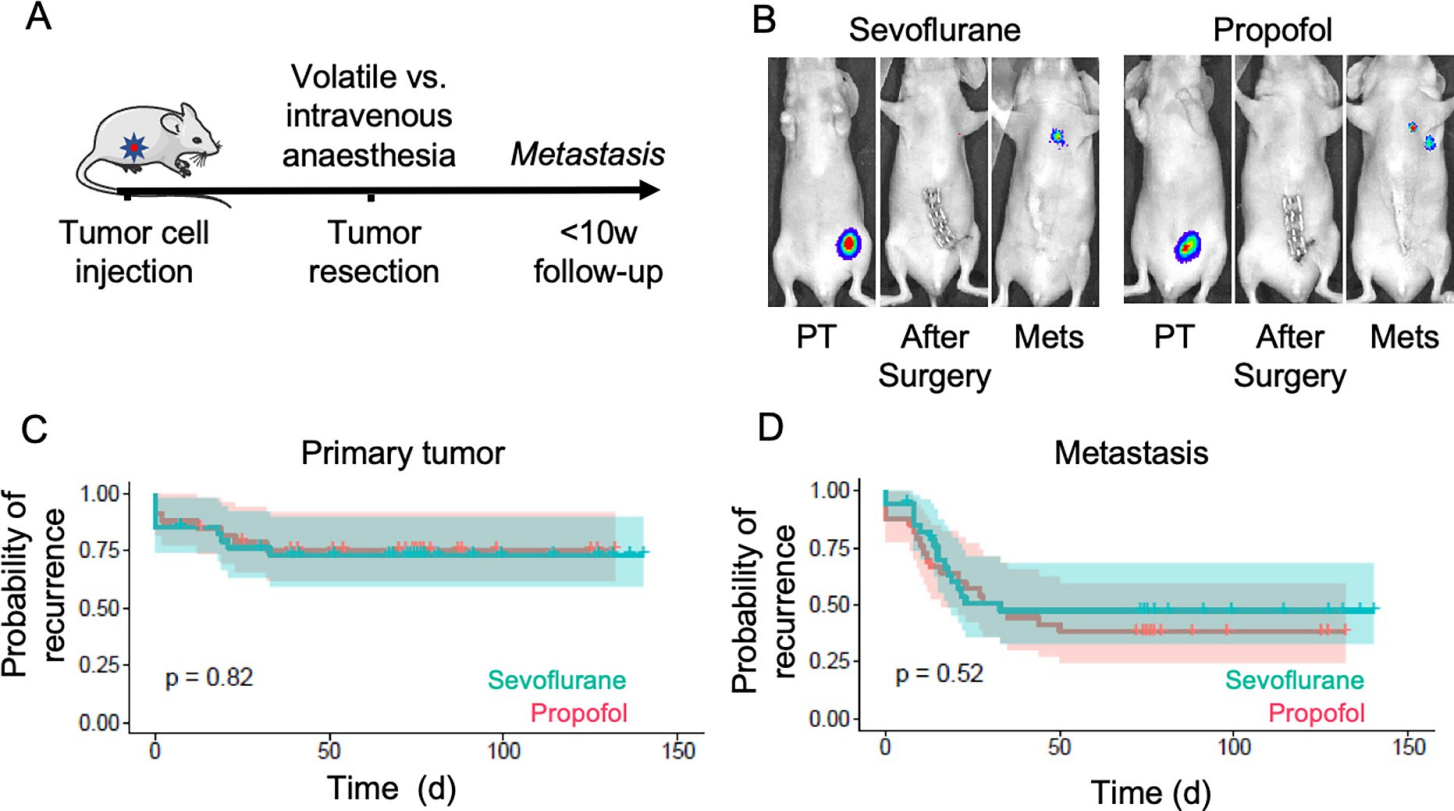
Cancer recurrence after surgical resection of MDA-MB-231 mammary tumors under sevoflurane versus propofol anaesthesia. A. Schematic of the experimental design. B. Bioluminescence was used to detect growth of MDA-MB-231 primary tumors (PT) in nude mice, demonstrate complete resection after surgery, and track onset and magnitude of recurrence at the primary site or metastatic target organs (Mets). C. Graph shows the probability of primary tumor recurrence after tumor resection. D. Graph shows the probability to onset of metastasis after primary tumor resection. Shaded area shows 95% confidence intervals. n = 33–34 mice per group. PT: primary tumor. Met: Metastasis.

To investigate if the choice of anesthetic agent used during cancer resection surgery impacts long-term cancer outcomes, we used bioluminescence imaging to track disease recurrence in mouse models of cancer (Fig 1A). MDA-MB-231 triple negative breast cancer cells were injected in the fourth mammary fatpad of Balb/c nude mice. When the mammary tumor reached 90mm^3^, mice were anesthetized with either sevoflurane or intravenous propofol and the tumor was resected. Bioluminescence imaging of luciferase-tagged tumor cells was used to confirm successful resection at the time of surgery, and to track onset and magnitude of local and distant recurrence after surgery (Fig 1B). Survival analysis investigated recurrence events pooled across experimental replicates (total: n = 33–34 per group, S1 Table (S1 File)) and found no statistical difference between the effects of volatile vs. intravenous anesthesia on time to primary tumor recurrence (Fig 1C), or time to metastasis (Fig 1D).

### Cancer recurrence in immune-competent mouse models of breast cancer

**Fig 2:**
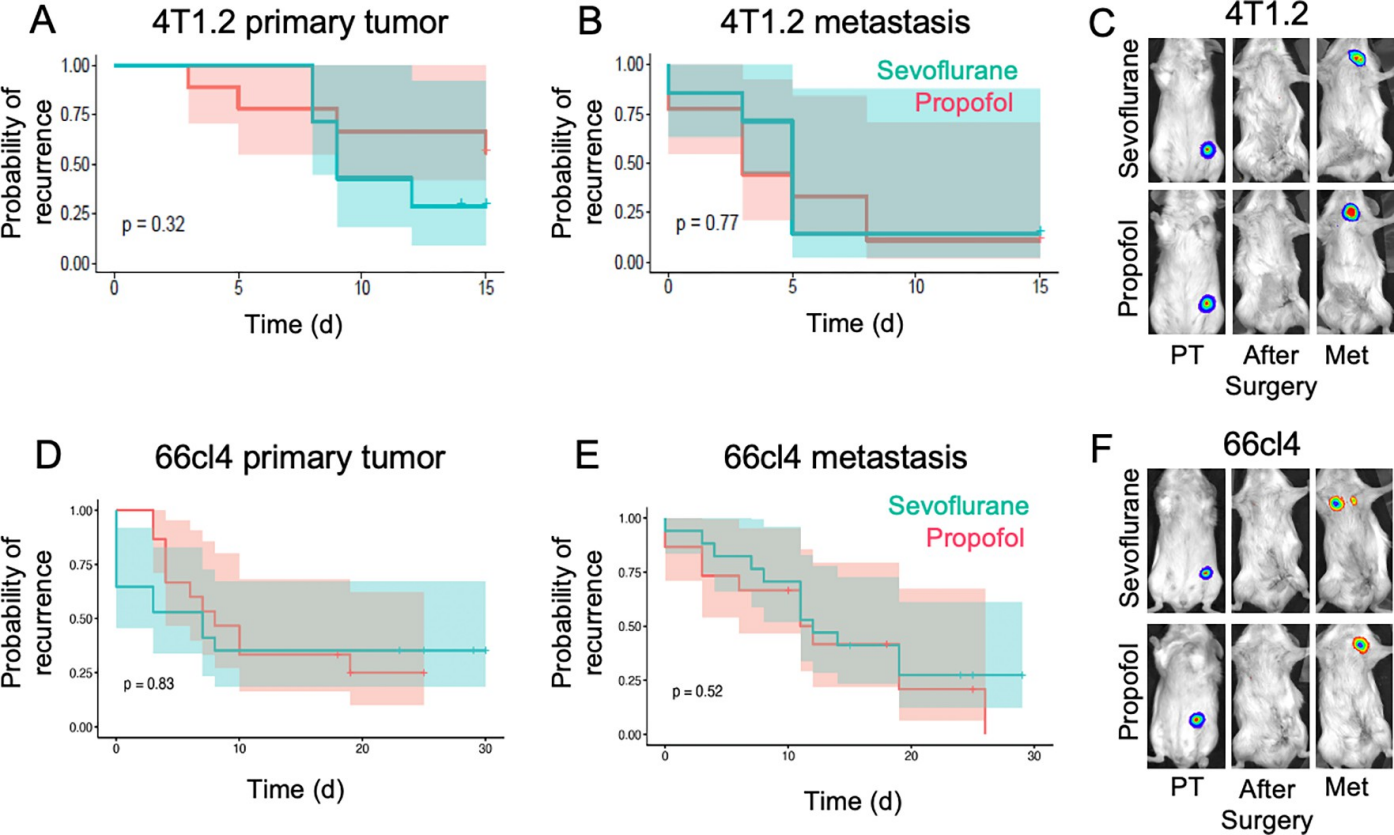
Cancer recurrence after surgical resection of mammary tumors from immune-intact mice under sevoflurane versus propofol anaesthesia. A and B. Graphs show the probability of (A) primary tumor recurrence and (B) metastasis development after 4T1.2 tumor resection. n = 7–9 mice per group. C. Representative bioluminescence images of mice prior to and after resection surgery and after metastasis development (Met). D, E. Graph shows the probability of (D) primary tumor recurrence and (E) metastasis after 66cl4 tumor resection. n = 15–17 mice per group. F. Representative bioluminescence images of mice prior and after surgery and after distant recurrence (metastasis). Shaded area shows 95% confidence intervals. PT: primary tumor. Met: Metastasis.

As volatile anesthesia has been suggested to modulate adaptive anti-cancer immunity [18, 24, 25], we hypothesized that a differential effect of anesthetic drug on recurrence may require an intact immune system. However, the human MDA-MB-231 model of breast cancer uses mice that are deficient in adaptive immunity to allow proliferation of human cancer cells. Therefore, we evaluated recurrence after primary tumor resection in two immune-competent mouse models of breast cancer. We first investigated recurrence after resection of 4T1.2 mammary tumors from Balb/c mice. This breast cancer model shows a high level of spontaneous metastasis from primary mammary tumors [33]. When the primary tumor became palpable it was resected under anesthesia using sevoflurane or intravenous propofol. Regardless of anesthesia type used, survival analyses found no difference in primary tumor recurrence or metastasis onset or progression (Fig 2A–2C). To investigate if the aggressive nature of this model (median time to metastasis: 5 days after surgery) obscured any effect of anesthesia on recurrence, we also investigated the effect of anesthesia in Balb/c mice with 66cl4 mammary tumors [33], a breast cancer model with slower onset of metastasis (median: 12 days after surgery). Again, survival analysis found that the type of anesthesia had no effect on the time to primary tumor or metastasis onset (Fig 2D–2F).

To increase the statistical power to identify an effect of anesthesia type, if one exists, we then undertook a Bayesian regression analysis that pooled results across all three cancer models, while accounting for differences between the models and variance within each model. The Bayesian regression analysis found that the credibility interval of the group effect estimate crossed zero, indicating that there was no difference in the time taken to primary tumor recurrence or time to onset of metastasis in mice that were anesthetized with sevoflurane compared to mice anesthetized with propofol (S2 Table (S1 File)). Furthermore, no evidence was found that anesthesia type impacted the kinetics of recurrence of either the primary tumor or metastasis (S2 Table (S1 File)). Therefore, using diverse analytical approaches (survival analysis, Bayesian regression analysis) we found no evidence to support the hypothesis that the choice of anesthetic agent used during cancer surgery impacts cancer recurrence in these mouse models of breast cancer.

### Effect of anesthesia on vascular integrity and innate immunity

**Fig 3:**
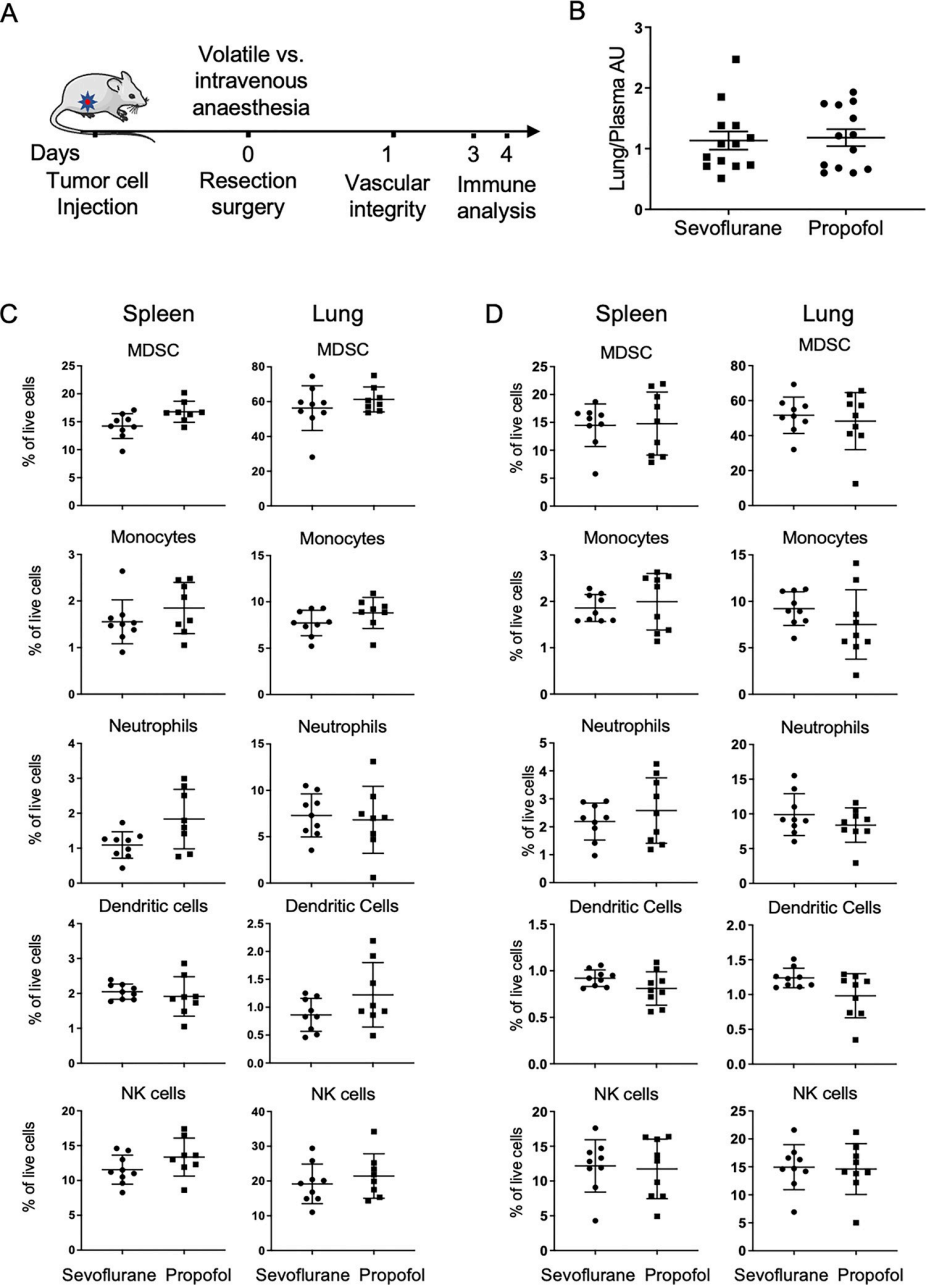
Vascular integrity and innate immune profiles after surgery with sevoflurane versus propofol anaesthesia. A. Schematic of the study design for mechanistic studies after resection of MDA-MB-231 mammary tumor under sevoflurane versus propofol anaesthesia. B. Lung vascular permeability was measured 24h after resection surgery by sodium fluorescein uptake into tissues. n = 13 per group, AU: arbitrary fluorescent units. C, D. Flow cytometric analysis of immune cells in spleen and lung on day 3 (C, n = 8–9 per group) and day 4 (D, n = 9 per group) after surgery. Data show mean ± SD. DC: dendritic cells, MDSC: myeloid derived suppressor cells, NK: natural killer cells.

While effects were not seen on cancer recurrence in these mouse models of breast cancer, we hypothesized that sevoflurane and intravenous propofol may have differential short-term effects on markers of stress and inflammation within the perioperative period (Fig 3A). As vascular integrity has been shown to be disrupted by perioperative events [34], we first explored the effect of anesthetic techniques on vascular permeability. Twenty-four hours after resection of MDA-MB-231 tumors, mice were injected systemically with fluorescein, which leaks into surrounding tissue when vascular integrity is lost. Quantification of tissue fluorescein levels showed no difference in vascular permeability after volatile anesthesia vs. propofol total intravenous anesthesia (p = 0.8) (Fig 3B).

In vitro studies have raised the possibility that anesthetic agents affect the immune response. Therefore, we examined if choice of anesthetic agent used for cancer resection surgery modulated innate immune cells in the spleen (a secondary lymphoid organ) at three days after surgery, when studies indicate that surgery-related inflammation is maximal [35, 36]. Flow cytometry profiling found no difference in percentage of myeloid-derived suppressor cells (MDSC), monocytes, neutrophils, natural killer cells or antigen-presenting dendritic cells in spleen at 3 days after surgery with anesthesia by propofol (Fig 3C). Similarly, we found no differences in immune cell populations in the lung, a key metastatic target, at three days after primary tumor resection with either sevoflurane or intravenous propofol (Fig 3C). We hypothesized that inflammation could plausibly mask any effect of anesthesia, therefore, we also profiled immune cells during the subsequent resolution phase [37–39]. Consistent with resolution of the immune response, percentages of some immune populations differed compared with day 3 after surgery and there was a suggestion of a reduction in dendritic cells in the lung (Fig 3D). However, statistical analyses found no differences in immune profiles of either spleen or lung in mice at 4 days after surgery with volatile anesthesia or intravenous propofol (Fig 3D).

### Effect of anesthesia on cellular immunity

**Fig 4:**
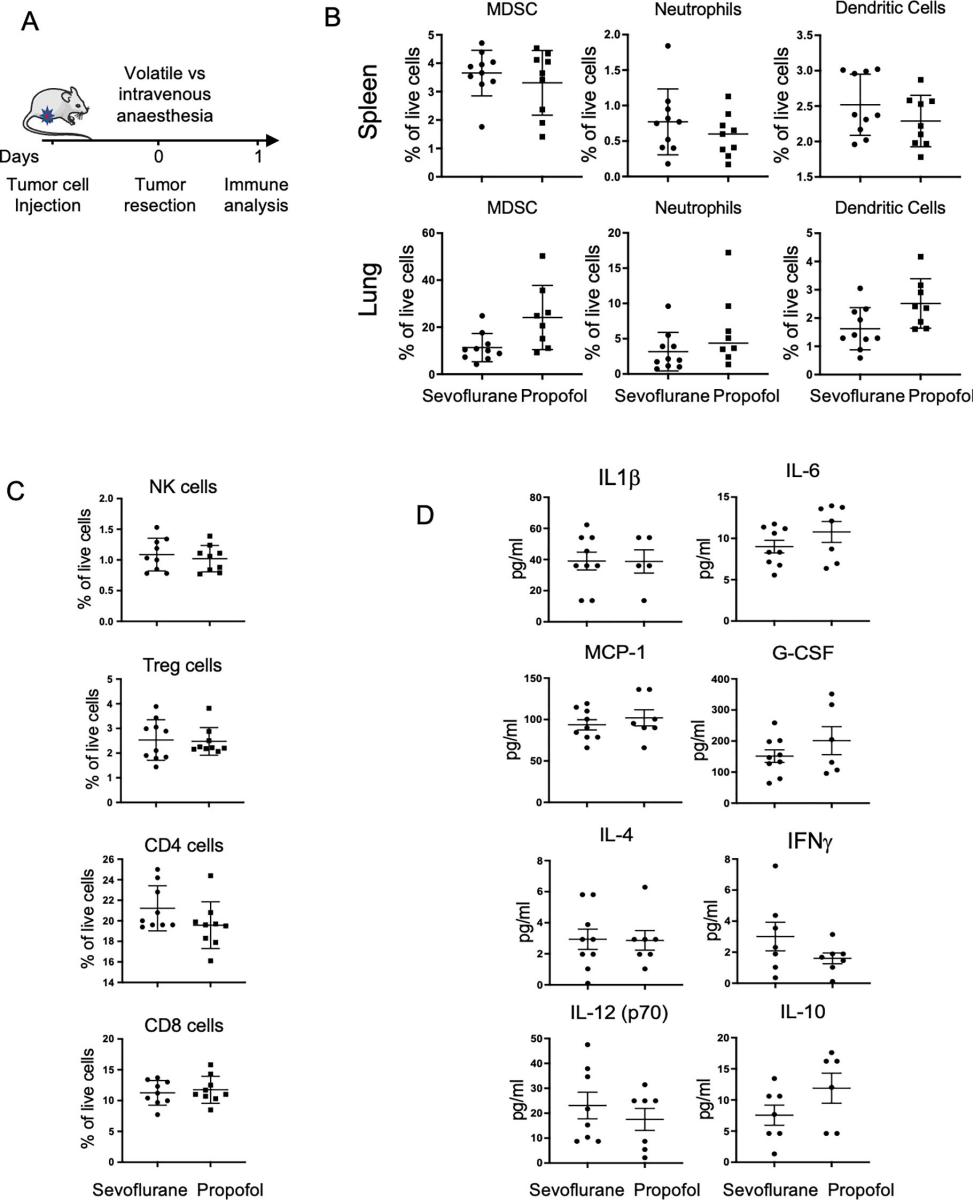
Innate and adaptive immune cells were profiled in lung and spleen after surgery with sevoflurane or propofol anaesthesia. A. Schematic of the experimental design for mechanistic studies after resection of 66cl4 tumor under sevoflurane versus propofol anaesthesia. B. Flow cytometry quantification of myeloid cells in spleen and lung. C. Flow cytometry analysis of lymphoid populations in spleen (n = 8–10 per group). D. Cytokine levels were measured in plasma using multiplex ELISA (n = 6–9 per group). Data show mean ± SD. G-CSF: granulocyte-colony stimulating factor, IFN: interferon, IL: Interleukin, MCP1: monocyte chemoattractant protein 1, NK: natural killer cell, Treg: T regulatory cell.

To evaluate the effect of anesthesia on immune profiles in the presence of a complete immune system, we investigated the effect of sevoflurane and intravenous propofol in Balb/c mice with 66cl4 mammary tumors (Fig 4A). As studies in MDA-MB-231 showed no effects at the peak or resolution of inflammation after surgery (3 and 4 days) [35, 36], we examined immune profiles at 24h after surgery. Analyses of flow cytometry quantification again found no difference in levels of immunosuppressive myeloid cells (MDSC), neutrophils or antigen-presenting dendritic cells in either the spleen or lung (Fig 4B), and no differences in natural killer or T cells in the spleen (Fig 4C). Finally, we hypothesized that cytokines may be a more sensitive indicator of inflammation than immune cell numbers, and so we assessed the effect of sevoflurane vs. intravenous propofol on plasma cytokine levels at 24h after surgery. Multiplex ELISA showed no effect of anesthetic technique on inflammatory (interleukin-1β, -6, -12) or anti-inflammatory cytokines (interleukin-10), or in cytokines involved in myeloid chemoattraction (e.g., monocyte chemoattractant protein 1, granulocyte-colony stimulating factor) or T cell polarization (interferon-γ and interleukin-4) (Fig 4D). Taken collectively, these studies in orthotopic mouse models of breast cancer demonstrated no differential effect of the type of anesthesia used during cancer resection surgery on either short-term or long-term cancer-related outcomes.

## Discussion

This series of preclinical studies found no evidence that the choice of anesthetic agent used during cancer resection surgery affects either short-term perioperative events or long-term recurrence outcomes in three mouse models of breast cancer. In contrast to retrospective clinical studies that suggest that use of volatile anesthesia is associated with reduced breast cancer survival [4, 8], here, the use of sevoflurane or propofol anesthesia during resection surgery resulted in similar patterns of primary tumor recurrence and metastatic recurrence in mouse models of breast cancer. Furthermore, we found no evidence that type of anesthesia modulated vascular integrity or immune response immediately after surgery. As a result, the findings do not currently support the notion that one type of anesthetic should be used in preference to the other.

The findings are limited by the extent to which mouse models replicate the clinical scenario experienced by breast cancer patients. As volatile anesthesia has been suggested to modulate adaptive anti-cancer immunity [18, 24, 25], we included mouse models with an intact immune system. Furthermore, as the site of tumor growth influences immune responses [40], we used orthotopic models to replicate tumor growth within mammary tissue (rather than flank tumors). Non-invasive bioluminescence imaging was used to confirm successful surgical resection and ensure sensitive detection of metastasis onset and progression. Nonetheless, it is possible that these models do not faithfully mirror the insult of cancer resection surgery. We speculated that a beneficial effect of propofol (if one exists) might depend on dampening an inflammatory response associated with surgery [19]. Mice are relatively resistant to inflammatory challenge compared to humans [41]. To account for this, we increased the inflammatory burden using invasive surgery at the time of primary tumor resection (S1 Fig S1 File). However, it is also plausible that an increased inflammatory insult at the time of surgery masked a differential effect of anesthesia type on cancer outcomes.

Other limitations of the experimental design included the brief use volatile anesthesia to insert the canula for delivery of intravenous anesthesia. Despite this, mice in the propofol condition remained under total intravenous propofol-based anesthesia for an hour but had less than 5 minutes exposure to sevoflurane. Additionally, exposure to volatile anesthesia for bioluminescence imaging during the post-surgical follow-up phase (twice per week) may have masked any effect of propofol-based TIVA. The use of postoperative analgesia (subcutaneous buprenorphine) was essential from a welfare consideration, but may have impacted postoperative inflammatory responses and cancer progression [42]. Buprenorphine was chosen for analgesia in these studies as its effects on the immune response are moderate compared with the marked immunosuppressive effects of other opioids [43]. Mu-opioid receptors have been documented on cancer cells, and receptor stimulation can increase cancer progression in preclinical models, although a large retrospective study found no increased risk of cancer or cancer-related mortality in opiate dependent patients receiving buprenorphine opioid replacement therapy [44]. Nonetheless, to minimize an effect of opioids on cancer progression, a consistent analgesia protocol was used across all conditions.

The results found here contrast with a recent study in 4T1 and MDA-MB-231 mouse models of breast cancer, which found that surgical resection of the primary mammary tumor under sevoflurane anesthesia led to more lung metastasis than resection with propofol [45]. Factors that might contribute to the contrasting findings include the strategy for propofol delivery, which was delivered by bolus intraperitoneal injection in that study [45], whereas our study delivered propofol by continuous low-dose infusion, which more closely resembles the approach used with cancer patients [46]. Furthermore, the contrasting findings may reflect the timing of primary tumor resection and anesthesia exposure relative to the onset of distant metastasis. In that study the primary tumor was resected at 500mm^3^ [45], by which time, in our hands, extensive metastasis has already occurred in these mouse models of breast cancer. Therefore, in the current study, the primary mammary tumor was resected earlier, prior to metastasis detection by non-invasive bioluminescence imaging, allowing us to define effects of earlier anaesthesia exposure on subsequent onset and magnitude of distant metastatic recurrence.

While these mechanistic studies were motivated by clinical findings [3, 4, 8], subsequent analyses in patients with breast cancer have also shown contradictory findings [9, 47]. A meta-analysis comparing anesthetic technique found that use of propofol TIVA anesthesia was associated with improved overall survival compared to volatile anesthesia (pooled HR, 0.76; 95% CI, 0.63–0.92; P < 0.01) [47]. However, the subgroup analyses of five breast cancer studies (four retrospective studies and one small prospective study) did not find a significant association of type of anesthesia used during section surgery with either recurrence free survival (HR 0.83; 95% CI, 0.59 to 1.15) or overall survival (HR, 1.12; 95% CI, 0.90 to 1.39) [47]. More recently, a retrospective study of 788 propensity-matched breast cancer patients found no difference in locoregional recurrence or overall survival with the use of propofol or desflurane anesthesia [48]. These findings raise the possibility that the type of cancer and invasiveness of surgery may be important factors in determining the impact of anesthetic technique on cancer outcomes. As such, tumor types other than breast cancer may be more suited to detection of an effect of anesthetic agent on cancer outcomes after surgery.

## Conclusions

The findings presented here suggest that the type of anesthesia used during cancer surgery had no impact either short-term or long-term cancer-related outcomes in mouse models of breast cancer. To better understand emerging retrospective clinical evidence in other cancer types, it will be important to investigate putative biological mechanisms in preclinical models as this will guide the design of future large-scale randomized clinical trials. If improved cancer outcomes can be achieved through the appropriate selection of anesthetic agent, it can be done for relatively little cost. While many of the new oncologic therapies are costed in thousands of dollars per patient, a change in anesthetic agent is costed in single dollar figures per patient. The importance of definitively identifying if anesthetic agents impact outcome in other cancer types, underpins the importance of the ongoing international multi-center VAPOR-C prospective clinical study, focused on lung and colorectal cancer (NCT04316013).

## Supporting information

S1 File Supporting information including S1 Fig, S1 and S2 Tables. (DOCX)

## References

[1] Sullivan R, Alatise OI, Anderson BO, Audisio R, Autier P, Aggarwal A, et al. Global cancer surgery: delivering safe, affordable, and timely cancer surgery. Lancet Oncol. 2015;16(11):1193–224. doi: 10.1016/S1470-2045(15)00223-5 . 26427363

[2] Lim A, Braat S, Hiller J, Riedel B. Inhalational versus propofol-based total intravenous anaesthesia: practice patterns and perspectives among Australasian anaesthetists. Anaesth Intensive Care. 2018;46(5):480–7. Epub 2018/09/08. doi: 10.1177/0310057X1804600509 . 30189822

[3] Wigmore TJ, Mohammed K, Jhanji S. Long-term Survival for Patients Undergoing Volatile versus IV Anesthesia for Cancer Surgery: A Retrospective Analysis. Anesthesiology. 2016;124(1):69–79. doi: 10.1097/ALN.0000000000000936 . 26556730

[4] Enlund M, Berglund A, Andreasson K, Cicek C, Enlund A, Bergkvist L. The choice of anaesthetic—sevoflurane or propofol—and outcome from cancer surgery: a retrospective analysis. Ups J Med Sci. 2014;119(3):251–61. doi: 10.3109/03009734.2014.922649 ; PubMed Central PMCID: PMC4116765. 24857018 PMC4116765

[5] Exadaktylos AK, Buggy DJ, Moriarty DC, Mascha E, Sessler DI. Can anesthetic technique for primary breast cancer surgery affect recurrence or metastasis? Anesthesiology. 2006;105(4):660–4. doi: 10.1097/00000542-200610000-00008 ; PubMed Central PMCID: PMC1615712. 17006061 PMC1615712

[6] Elias KM, Kang S, Liu X, Horowitz NS, Berkowitz RS, Frendl G. Anesthetic selection and disease-free survival following optimal primary cytoreductive surgery for stage III epithelial ovarian cancer. Ann Surg Oncol. 2015;22(4):1341–8. doi: 10.1245/s10434-014-4112-9 . 25287437

[7] Melchi CF, Mele A, Baliva G, Scio M, Fucci M, Pasquini P, et al. Prognostic value of anesthesia type for patients treated for cutaneous melanoma. Dermatol Surg. 1995;21(9):786–8. doi: 10.1111/j.1524-4725.1995.tb00297.x . 7655798

[8] Lee JH, Kang SH, Kim Y, Kim HA, Kim BS. Effects of propofol-based total intravenous anesthesia on recurrence and overall survival in patients after modified radical mastectomy: a retrospective study. Korean J Anesthesiol. 2016;69(2):126–32. doi: 10.4097/kjae.2016.69.2.126 ; PubMed Central PMCID: PMC4823406. 27066202 PMC4823406

[9] Karami MY, Dehghanpisheh L, Karami A, Sabzloun Z, Niazkar HR, Mojarad N, et al. Comparison of volatile/inhalational and IV anesthesia in long-term survival of patients with breast cancer: a retrospective study. Eur J Med Res. 2022;27(1):271. Epub 2022/12/04. doi: 10.1186/s40001-022-00911-9 ; PubMed Central PMCID: PMC9719258. 36463276 PMC9719258

[10] Weitz J, Kienle P, Lacroix J, Willeke F, Benner A, Lehnert T, et al. Dissemination of tumor cells in patients undergoing surgery for colorectal cancer. Clin Cancer Res. 1998;4(2):343–8. . 9516921

[11] Luzzi KJ, MacDonald IC, Schmidt EE, Kerkvliet N, Morris VL, Chambers AF, et al. Multistep nature of metastatic inefficiency: dormancy of solitary cells after successful extravasation and limited survival of early micrometastases. The American journal of pathology. 1998;153(3):865–73. Epub 1998/09/15. doi: 10.1016/S0002-9440(10)65628-3 ; PubMed Central PMCID: PMC1853000. 9736035 PMC1853000

[12] Benzonana LL, Perry NJ, Watts HR, Yang B, Perry IA, Coombes C, et al. Isoflurane, a commonly used volatile anesthetic, enhances renal cancer growth and malignant potential via the hypoxia-inducible factor cellular signaling pathway in vitro. Anesthesiology. 2013;119(3):593–605. doi: 10.1097/ALN.0b013e31829e47fd . 23774231

[13] Iwasaki M, Zhao H, Jaffer T, Unwith S, Benzonana L, Lian Q, et al. Volatile anaesthetics enhance the metastasis related cellular signalling including CXCR2 of ovarian cancer cells. Oncotarget. 2016;7(18):26042–56. doi: 10.18632/oncotarget.8304 ; PubMed Central PMCID: PMC5041963. 27028996 PMC5041963

[14] Liu Z, Zhang J, Hong G, Quan J, Zhang L, Yu M. Propofol inhibits growth and invasion of pancreatic cancer cells through regulation of the miR-21/Slug signaling pathway. Am J Transl Res. 2016;8(10):4120–33. ; PubMed Central PMCID: PMC5095306. 27829997 PMC5095306

[15] Melamed R, Rosenne E, Shakhar K, Schwartz Y, Abudarham N, Ben-Eliyahu S. Marginating pulmonary-NK activity and resistance to experimental tumor metastasis: suppression by surgery and the prophylactic use of a beta-adrenergic antagonist and a prostaglandin synthesis inhibitor. Brain Behav Immun. 2005;19(2):114–26. doi: 10.1016/j.bbi.2004.07.004 . 15664784

[16] Acharya NK, Goldwaser EL, Forsberg MM, Godsey GA, Johnson CA, Sarkar A, et al. Sevoflurane and Isoflurane induce structural changes in brain vascular endothelial cells and increase blood-brain barrier permeability: Possible link to postoperative delirium and cognitive decline. Brain Res. 2015;1620:29–41. doi: 10.1016/j.brainres.2015.04.054 . 25960348

[17] Woods GM, Griffiths DM. Reversible inhibition of natural killer cell activity by volatile anaesthetic agents in vitro. Br J Anaesth. 1986;58(5):535–9. doi: 10.1093/bja/58.5.535 . 3457589

[18] Stollings LM, Jia LJ, Tang P, Dou H, Lu B, Xu Y. Immune Modulation by Volatile Anesthetics. Anesthesiology. 2016;125(2):399–411. doi: 10.1097/ALN.0000000000001195 ; PubMed Central PMCID: PMC5074538. 27286478 PMC5074538

[19] Hiller JG, Perry NJ, Poulogiannis G, Riedel B, Sloan EK. Perioperative events influence cancer recurrence risk after surgery. Nat Rev Clin Oncol. 2018;15(4):205–18. Epub 2017/12/29. doi: 10.1038/nrclinonc.2017.194 . 29283170

[20] Chen RM, Chen TG, Chen TL, Lin LL, Chang CC, Chang HC, et al. Anti-inflammatory and antioxidative effects of propofol on lipopolysaccharide-activated macrophages. Ann N Y Acad Sci. 2005;1042:262–71. doi: 10.1196/annals.1338.030 . 15965071

[21] Le CP, Nowell CJ, Kim-Fuchs C, Botteri E, Hiller JG, Ismail H, et al. Chronic stress in mice remodels lymph vasculature to promote tumour cell dissemination. Nature communications. 2016;7:10634. doi: 10.1038/ncomms10634 ; PubMed Central PMCID: PMC4773495. 26925549 PMC4773495

[22] Chang A, Le CP, Walker AK, Creed SJ, Pon CK, Albold S, et al. Beta2-adrenoceptors on tumor cells play a critical role in stress-enhanced metastasis in a mouse model of breast cancer. Brain Behav Immun. 2016;57:106–15. doi: 10.1016/j.bbi.2016.06.011 . 27321906 PMC5060133

[23] Sloan EK, Priceman SJ, Cox BF, Yu S, Pimentel MA, Tangkanangnukul V, et al. The sympathetic nervous system induces a metastatic switch in primary breast cancer. Cancer Res. 2010;70(18):7042–52. Epub 2010/09/09. doi: 10.1158/0008-5472.CAN-10-0522 ; PubMed Central PMCID: PMC2940980. 20823155 PMC2940980

[24] Wall T, Sherwin A, Ma D, Buggy DJ. Influence of perioperative anaesthetic and analgesic interventions on oncological outcomes: a narrative review. Br J Anaesth. 2019;123(2):135–50. Epub 2019/07/01. doi: 10.1016/j.bja.2019.04.062 ; PubMed Central PMCID: PMC6676329. 31255291 PMC6676329

[25] Lim JA, Oh CS, Yoon TG, Lee JY, Lee SH, Yoo YB, et al. The effect of propofol and sevoflurane on cancer cell, natural killer cell, and cytotoxic T lymphocyte function in patients undergoing breast cancer surgery: an in vitro analysis. BMC Cancer. 2018;18(1):159. Epub 2018/02/09. doi: 10.1186/s12885-018-4064-8 ; PubMed Central PMCID: PMC5803927. 29415668 PMC5803927

[26] Uphoff CC, Drexler HG. Comparative PCR analysis for detection of mycoplasma infections in continuous cell lines. In Vitro Cell Dev Biol Anim. 2002;38(2):79–85. Epub 2002/04/04. doi: 10.1290/1071-2690(2002)038&lt;0079:CPAFDO&gt;2.0.CO;2 . 11928999

[27] Kaminskas LM, Ascher DB, McLeod VM, Herold MJ, Le CP, Sloan EK, et al. PEGylation of interferon alpha2 improves lymphatic exposure after subcutaneous and intravenous administration and improves antitumour efficacy against lymphatic breast cancer metastases. Journal of controlled release: official journal of the Controlled Release Society. 2013;168(2):200–8. doi: 10.1016/j.jconrel.2013.03.006 ; PubMed Central PMCID: PMC4022972. 23499718 PMC4022972

[28] Seok J, Warren HS, Cuenca AG, Mindrinos MN, Baker HV, Xu W, et al. Genomic responses in mouse models poorly mimic human inflammatory diseases. Proc Natl Acad Sci U S A. 2013;110(9):3507–12. doi: 10.1073/pnas.1222878110 ; PubMed Central PMCID: PMC3587220. 23401516 PMC3587220

[29] Shaashua L, Rosenne E, Neeman E, Sorski L, Sominsky L, Matzner P, et al. Plasma IL-12 levels are suppressed in vivo by stress and surgery through endogenous release of glucocorticoids and prostaglandins but not catecholamines or opioids. Psychoneuroendocrinology. 2014;42:11–23. Epub 2014/03/19. doi: 10.1016/j.psyneuen.2013.12.001 ; PubMed Central PMCID: PMC3959722. 24636497 PMC3959722

[30] Therneau T. A Package for Survival Analysis in R 2020. R package version 3.2–7]. Available from: https://CRAN.R-project.org/package=survival.

[31] Bürkner P-C. brms: An R Package for Bayesian Multilevel Models Using Stan. Journal of Statistical Software. 2017;80(1). doi: 10.18637/jss.v080.i01

[32] R CT. R: A language and environment for statistical computing: R Foundation for Statistical Computing, Vienna, Austria.; 2020. Available from: https://www.R-project.org/.

[33] Eckhardt BL, Parker BS, van Laar RK, Restall CM, Natoli AL, Tavaria MD, et al. Genomic analysis of a spontaneous model of breast cancer metastasis to bone reveals a role for the extracellular matrix. Mol Cancer Res. 2005;3(1):1–13. . 15671244

[34] Riedel B, Schier R. Endothelial dysfunction in the perioperative setting. Seminars in cardiothoracic and vascular anesthesia. 2010;14(1):41–3. doi: 10.1177/1089253210362793 . 20472622

[35] Coffey JC, Wang JH, Smith MJ, Bouchier-Hayes D, Cotter TG, Redmond HP. Excisional surgery for cancer cure: therapy at a cost. Lancet Oncol. 2003;4(12):760–8. Epub 2003/12/10. doi: 10.1016/s1470-2045(03)01282-8 . 14662433

[36] Ogawa K, Hirai M, Katsube T, Murayama M, Hamaguchi K, Shimakawa T, et al. Suppression of cellular immunity by surgical stress. Surgery. 2000;127(3):329–36. Epub 2000/03/15. doi: 10.1067/msy.2000.103498 . 10715990

[37] Park JE, Barbul A. Understanding the role of immune regulation in wound healing. Am J Surg. 2004;187(5A):11S–6S. Epub 2004/05/19. doi: 10.1016/S0002-9610(03)00296-4 . 15147986

[38] Tsirogianni AK, Moutsopoulos NM, Moutsopoulos HM. Wound healing: immunological aspects. Injury. 2006;37 Suppl 1:S5–12. Epub 2006/04/18. doi: 10.1016/j.injury.2006.02.035 . 16616753

[39] Tang F, Tie Y, Tu C, Wei X. Surgical trauma-induced immunosuppression in cancer: Recent advances and the potential therapies. Clin Transl Med. 2020;10(1):199–223. Epub 2020/06/09. doi: 10.1002/ctm2.24 ; PubMed Central PMCID: PMC7240866. 32508035 PMC7240866

[40] Devaud C, Westwood JA, John LB, Flynn JK, Paquet-Fifield S, Duong CP, et al. Tissues in different anatomical sites can sculpt and vary the tumor microenvironment to affect responses to therapy. Molecular therapy: the journal of the American Society of Gene Therapy. 2014;22(1):18–27. doi: 10.1038/mt.2013.219 ; PubMed Central PMCID: PMC3978809. 24048441 PMC3978809

[41] Schaedler RW, Dubos RJ. The susceptibility of mice to bacterial endotoxins. J Exp Med. 1961;113:559–70. Epub 1961/03/01. doi: 10.1084/jem.113.3.559 ; PubMed Central PMCID: PMC2137366. 13747161 PMC2137366

[42] Al-Hashimi M, Scott SWM, Thompson JP, Lambert DG. Opioids and immune modulation: more questions than answers. British Journal of Anaesthesia. 2013;111(1):80–8. doi: 10.1093/bja/aet153 23794649

[43] DeMarco GJ, Nunamaker EA. A Review of the Effects of Pain and Analgesia on Immune System Function and Inflammation: Relevance for Preclinical Studies. Comp Med. 2019;69(6):520–34. Epub 2020/01/04. doi: 10.30802/AALAS-CM-19-000041 ; PubMed Central PMCID: PMC6935697. 31896389 PMC6935697

[44] Kelty E, Dobbins T, Hulse G. Incidence of cancer and cancer related mortality in opiate dependent patients treated with methadone, buprenorphine or implant naltrexone as compared with non-opiate using controls. Heroin Addiction and Related Clinical Problems. 2017;19(3):65–72.

[45] Li R, Huang Y, Lin J. Distinct effects of general anesthetics on lung metastasis mediated by IL-6/JAK/STAT3 pathway in mouse models. Nature communications. 2020;11(1):642. Epub 2020/02/02. doi: 10.1038/s41467-019-14065-6 ; PubMed Central PMCID: PMC6994546. 32005799 PMC6994546

[46] Dubowitz JA, Jost-Brinkmann F, Ziegler AI, Gillis RD, Riedel B, Sloan EK. An In Vivo Mouse Model of Total Intravenous Anesthesia during Cancer Resection Surgery. J Vis Exp. 2021;(172). Epub 2021/06/29. doi: 10.3791/62747 . 34180906

[47] Yap A, Lopez-Olivo MA, Dubowitz J, Hiller J, Riedel B. Anesthetic technique and cancer outcomes: a meta-analysis of total intravenous versus volatile anesthesia. Canadian journal of anaesthesia=Journal canadien d’anesthesie. 2019. Epub 2019/03/06. doi: 10.1007/s12630-019-01330-x . 31218535

[48] Huang Y-H, Lee M-S, Lou Y-S, Lai H-C, Yu J-C, Lu C-H, et al. Propofol-based total intravenous anesthesia did not improve survival compared to desflurane anesthesia in breast cancer surgery. PLOS ONE. 2019;14(11):e0224728. doi: 10.1371/journal.pone.0224728 31697743 PMC6837387

