# Supplementary material for: Type of anesthesia for cancer resection surgery: No differential impact on cancer recurrence in mouse models of breast cancer
Source: PLoS One. 2023 Nov 27;18(11):e0293905. doi: 10.1371/journal.pone.0293905 (PMC10681249; doi:10.1371/journal.pone.0293905)
Supplement: S1 File — (DOCX) [file pone.0293905.s001.docx]

**Supporting information for**

Type of anesthesia for cancer resection surgery:

No differential impact on cancer recurrence in mouse models of breast cancer

Julia Dubowitz, Alexandra I. Ziegler, Richard Beare,

Fabian Jost-Brinkman, Adam K. Walker, Ryan D. Gillis, Aeson Chang, Ni-Chun Chung, Olga Martin, Frédéric Hollande, Bernhard Riedel, Erica K. Sloan

**Supplementary Figure 1.** **Inflammatory response induced by resection surgery.** Resection of the mammary tumor with laparotomy (major surgery) increased the inflammatory response as seen by increased spleen mass and increased cytokine transcription, compared to unoperated tumor-bearing mice (control) or mice after resection of only the mammary tumor (minor surgery). * P < .04 by one-way ANOVA.

**Supplementary Table 1. Pooled recurrence events.** Pooled recurrence events for each breast cancer model.

| Breast cancer model | Anaesthetic type | Total mice | Primary tumor recurrence  Number (percentage) | Metastasis  Number (percentage) |
| --- | --- | --- | --- | --- |
| MDA-MB-231 | Sevoflurane | 34 | 9 (26%) | 17 (50%) |
|  | Propofol | 33 | 8 (24%) | 20 (61%) |
| 66cl4 | Sevoflurane | 17 | 11 (65%) | 12 (71%) |
|  | Propofol | 15 | 11 (73%) | 11 (73%) |
| 4T1.2 | Sevoflurane | 7 | 5 (71%) | 6 (86%) |
|  | Propofol | 9 | 4 (44%) | 8 (89%) |

**Supplementary Table 2.** **Difference in beta coefficients.** Difference in beta coefficients between anesthesia treatment groups (sevoflurane minus propofol) estimated by Bayesian multilevel regression, and 95% credibility intervals (CI).

|  | Primary tumor | Metastasis |
| --- | --- | --- |
| Survival time | -0.41 (CI = -1.53, 0.70) | 0.39 (CI = -0.26, 1.08) |
| Recurrence kinetics (log flux) | 0.00 (CI = -0.04, 0.04) | 0.02 (CI = -0.02, 0.05) |
